# Survival Rates of Extremely Low-Birth-Weight Infants in a Tertiary Care Center in Saudi Arabia

**DOI:** 10.7759/cureus.54462

**Published:** 2024-02-19

**Authors:** Mohammad Alhasoon

**Affiliations:** 1 Department of Pediatrics, College of Medicine, Qassim University, Unaizah, SAU

**Keywords:** kingdom of saudi arabia (ksa), elbw neonates, survival rates, neonates, extremely low birth weight

## Abstract

Introduction

Extremely low birth weight (ELBW) refers to the condition in which an infant is born with a weight of less than one thousand grams (2.2 pounds) at birth. ELBW infants face significant challenges and are at increased risk for various medical complications and developmental issues. ELBW poses unique challenges for infants, families, and healthcare providers. Understanding the causes, consequences, and appropriate management strategies for ELBW is crucial for improving the survival rates of these vulnerable infants.

Aim

This study aimed to measure the survival rates of ELBW infants in Saudi Arabia and its correlated risk factors.

Patients and methods

This case-control study was a retrospective chart review analysis of data from King Abdulaziz Medical City (KAMC), a single tertiary care center in Riyadh, Kingdom of Saudi Arabia, and conducted over a four-year period. To estimate the survival rate among all live-birth newborn infants who were born with ELBWs of less than 1000 grams, collected data were tabulated and cleaned in MS Excel, and all data were analyzed using IBM SPSS Statistics for Windows, version 26 (released 2019; IBM Corp., Armonk, New York, United States).

Results

Two hundred and fifty-six patients were involved. Non-survival rates were 12.9%. In a multivariate regression model, prolonged rupture of membranes (PROM), periventricular leukomalacia (PVL), major intraventricular hemorrhage (IVH), and longer length of stay had increased risks for non-survival, while increasing gestational age, APGAR scores, and cesarean section had decreased risks for non-survival. Survival analysis found that there was a significant mean difference in gestational age (weeks) survival time between normal spontaneous vaginal delivery (NSVD) and cesarean section based on log-rank (Mantel-Cox) (p = 0.008).

Conclusion

Consistent with the literature, a greater prevalence of ELBW infants survived during hospital stay. Independent risk factors for non-survival include PROM, PVL, major IVH, and long length of stay. Cesarean section, increasing gestational, and APGAR scores were identified as the independent predictors of survival. Prospective studies in nature are required to determine these factors' cause and effect.

## Introduction

Extremely low birth weight (ELBW) refers to the condition in which an infant is born with a weight of less than one thousand grams (2.2 pounds) at birth. ELBW infants face significant challenges and are at increased risk for various medical complications and developmental issues. ELBW poses unique challenges for infants, families, and healthcare providers. Understanding the causes, consequences, and appropriate management strategies for ELBW is crucial for improving the survival rates of these vulnerable infants.

Globally, ELBW infants represent a total of 1.2 to 1.5 percent of all live births and 15 to 20 percent of neonatal intensive care unit (NICU) admissions [1]. Aside from how prematurity accounts for approximately 11.1% of all pregnancies and deliveries, the global survival rates have increased sharply over the last five decades [2,3]. Advances in neonatal care assisted with decreasing the morbidity and mortality rates. However, the high prevalence of ELBW in Saudi Arabia presents considerable mortality risks when more than one-third of prematurely born infants admitted to NICUs die before they are discharged [4]. Infants with ELBW frequently endure health complications, such as intraventricular hemorrhage (IVH), yet parents may decide to initiate or withdraw life support when the likelihood of viability is questionable [5,6]. 

Concurrently, mothers giving birth to premature infants present regional variations indicating that social, environmental, and economic factors inform neonatal care decisions [7,8]. These variations receive little to no scholarly attention and support the recommendation that researchers must obtain more survey or interview data when addressing which determinants impact ELBWs in Saudi Arabia.

The research literature highlights regional variations in survival rates among ELBW infants. Differences between the early and late outcomes of ELBW infants in Central Saudi Arabia received scholarly attention when researchers noted the onset of cognitive and neurodevelopmental abnormalities. Although newborns before 23 to 25 weeks of gestation are minimally viable, mothers in Saudi Arabia receive little obstetrical support to address what causes the epidemiological problem. Studies conducted in the Jazan region of Saudi Arabia have found that pregnancy complications and demographic factors contribute to LBWs in mothers. Research done at academic hospitals has also shown that ELBW infants often experience different complications, and improvements in survival rates have been limited.

The problem informing this proposed study concerns how few studies provide reliable quantitative data emphasizing the survival rates of ELBW infants in Saudi Arabia. One recently published study accounted for the variations in survival rates from 1994 to 2019 [1]. However, few current studies address that targeted preterm neonatal intervention strategies effectively mitigate the problem or decrease prevalence rates [7]. Healthcare leaders may identify performance benchmarks whereby obstetricians continuously monitor for changes in morbidity and mortality rates at local institutions. However, researchers have to fully document the extent of prematurity causing ELBW while monitoring for short-term and long-term health outcomes [3,8]. Considering how the problem typically results in serious health complications following delivery, its epidemiological implications are such that clinical researchers need more empirical data evaluating which factors affect how women respond [4]. Similarly, the dearth of scholarship demonstrates a need for researchers to offer evidence-based recommendations for improved clinical decision-making [5]. Filling these lacunae demands closer attention to local and regional variations in ELBW prevalence rates. Collecting survey or interview data to document how women in Saudi Arabia address prematurity and mortality risks is also necessary.

## Materials and methods

This case-control study spanned four years, from January 2017 to December 2020, and was carried out at King Abdulaziz Medical City (KAMC), Riyadh, Kingdom of Saudi Arabia. With an average of 2,300 admissions annually, the Neonatal Intensive Care Unit (NICU) at KAMC consists of a 36-bed intermediate care nursery and a 40-bed level III critical care unit.

ELBWs at all the research comprised newborns born at KAMC with birth weights under 1,000 grams. The study eliminated infants who died out of the womb and those who had fatal defects. The institutional review board of King Abdullah International Medical Research Centre (KAIMRC) accepted the study (IRB: RC20/283/R) and waived the need for patient permission because the study was a retrospective chart review and no personally identifiable patient data was retained in the database.

The analysis was restricted to women who delivered a live infant either singleton or multiple births at 23 or more weeks. ELBW infants were defined as those born weighing less than 1,000 g, and very low birth weight (VLBW) infants were defined as those weighing less than 1,500 g.

A total of 256 ELBW infants born at 23-37 weeks of gestation were enrolled over four years in the period between January 2017 and December 2020. A case was defined as death at discharge. The other cases, that is, survival at discharge, were used as controls.

The prenatal and inpatient records were analyzed to extract demographic information on mothers and their newborns. Gestational age, chorionicity, and amnionicity were determined from prenatal records. For an uncertain last menstrual period, gestational age from the earliest ultrasound scan was used to determine the gestational age.

More than 10 variables were assessed, including demographic data and prenatal events. Maternal age, the number of fetuses, the prenatal use of corticosteroids, the presence of gestational diabetes and hypertension, the mode of delivery, gender, birthweight, gestational age at birth, and APGAR score were among the maternal and neonatal variables that were examined for the population description. Prolonged rupture of membranes (PROM), which is defined as "rupture of membranes more than 18 hours before delivery," was also examined. The following neonatal morbidities were assessed in order to determine whether they contributed to the non-survival: surgical necrotizing enterocolitis (NEC) that needed surgical intervention with insertion of intraabdominal drain or laparotomy, retinopathy of prematurity (ROP) based on pediatric ophthalmologic examination using the international classification of ROP that needed intravitreal injection therapy and/or laser photocoagulation, intraventricular hemorrhage (IVH) (grades III and IV) based on the Papile classification, cystic or diffuse periventricular leukomalacia (PVL) by ultrasound and/or MRI, and culture-positive sepsis of the blood, CSF, or urine. The number of days that the patient was on mechanical ventilation and the length of hospital stay until they were allowed to go home were also calculated.

Ethical approval and informed consent

The study was authorized by the KAIMRC under the IRB code RC20/283/R. The study was conducted in compliance with the Declaration of Helsinki, and all procedures were carried out in compliance with applicable laws and guidelines. This was a case-control study with a review of the charts and no personally identifying patient data. The ethical committee decided not to require patient consent.

Statistical analysis

The data were analyzed using the software program IBM SPSS Statistics for Windows, version 26 (released 2019; IBM Corp., Armonk, New York, United States). Descriptive statistics were given as numbers and percentages (%) for all categorical variables, while continuous variables were calculated and summarized as mean and standard deviation. The relationship between the overall outcome and patients' characteristics was conducted using the chi-square test. Significant results were then gathered in univariate and multivariate regression models to determine the independent risk factors associated with non-survival rates with corresponding odds ratios (ORs) and 95% confidence intervals (CIs). In addition, we performed survival analyses between the outcome (alive vs. death) in association with the gender and method of delivery, measuring their survival time according to their length of stay and gestational age. Values were considered significant with a p-value of less than 0.05.

## Results

This study analyzed 256 patients. As shown in Table 1, 56.6% of the infants were males. The mean gestational age was 29.1 weeks, with 60.5% categorized between 30 and 33 weeks. The mean birth weight of infants was 773, and 57.4% were considered to have a 750 g or higher birth weight. The most common method of delivery was cesarean section (80.1%). Maternal hypertension (HTN), diabetes, and PROM constitute 10.5%, 15.6%, and 17.6%, respectively. Completed steroid use constituted 73%. Chorionicity was mostly dizygotic dichorionic diamniotic (DC DA) (44.5%). More than half (54.7%) were spontaneous pregnancy. PVL, major IVH, ROP that needs intervention, surgical necrotizing enterocolitis, bronchopulmonary dysplasia, and culture-positive sepsis constituted 8.6%, 14.5%, 10.2%, 5.9%, 28.5%, and 23.8%, respectively. The number of fetuses was mainly triplets (57.8%). The mean duration of ventilation was 9.76 days, while the length of hospital stays was 46.7 days. The maternal mean age was 30.3 years. The mean parity was 1.75. The mean Apgar scores for one minute and five minutes were 5.94 and 7.76, respectively.

**Table 1:** Outcomes of ELBW infants according to the patient's characteristics § P-value has been calculated using the chi-square test. ‡ P-value has been calculated using the independent sample t-test. ** Significant at p < 0.05 level. DC DA: dichorionic diamniotic, MC DA: monochorionic diamniotic, MC MA: monochorionic monoamniotic, ELBW: extremely low birth weight, NSVD: normal spontaneous vaginal delivery, HTN: hypertension, PROM: prolonged rupture of membranes, ROP: retinopathy of prematurity

Study variables	Overall N (%) ^(n=256)^	Outcome		P-value ^§^
		Death N (%) ^(n=33)^	Alive N (%) ^(n=223)^	
Gender				
Male	145 (56.6%)	16 (48.5%)	129 (57.8%)	0.311
Female	111 (43.4%)	17 (51.5%)	94 (42.2%)	
Gestational age				
22-26 weeks	58 (22.7%)	20 (60.6%)	38 (17.0%)	<0.001 **
27-29 weeks	43 (16.8%)	05 (15.2%)	38 (17.0%)	
30-33 weeks	155 (60.5%)	08 (24.2%)	147 (65.9%)	
Mean ± SD	29.1 ± 2.94	26.2 ± 3.35	29.5 ± 2.62	<0.001 **
Birth weight				
<1000 g	147 (57.4%)	24 (72.7%)	123 (55.2%)	0.057
<750 g	109 (42.6%)	09 (27.3%)	100 (44.8%)	
Mean ± SD	773.0 ± 155.4	851.5 ± 157.5	761.4 ± 152.1	0.002 **
Mode of delivery				
NSVD	51 (19.9%)	11 (33.3%)	40 (17.9%)	0.039 **
Cesarean section	205 (80.1%)	22 (66.7%)	183 (82.1%)	
Maternal factor				
Maternal HTN/preeclampsia	27 (10.5%)	01 (97.0%)	26 (11.7%)	0.220
Maternal diabetes	40 (15.6%)	06 (18.2%)	34 (15.2%)	0.665
PROM (>18 hours)	45 (17.6%)	10 (30.3%)	35 (15.7%)	0.040 **
Antenatal steroid use				
No	32 (12.5%)	02 (06.1%)	30 (13.5%)	0.293
Incomplete	37 (14.5%)	07 (21.2%)	30 (13.5%)	
Complete	187 (73.0%)	24 (72.7%)	163 (73.1%)	
Chorionicity			
Dizygotic DC DA*	114 (44.5%)	09 (27.3%)	105 (47.1%)	0.368
Monozygotic DC DA	38 (14.8%)	07 (21.2%)	31 (13.9%)	
Monozygotic MC DA*	03 (01.2%)	0	03 (01.3%)	
Monozygotic MC MA*	56 (21.9%)	08 (24.2%)	48 (21.5%)	
Triplets	12 (04.7%)	03 (09.1%)	09 (04.0%)	
Quintuplets	06 (02.3%)	01 (03.0%)	05 (02.2%)	
Spontaneous pregnancy or assisted conception				
Spontaneous	140 (54.7%)	15 (45.5%)	125 (56.1%)	0.254
Assisted	116 (45.3%)	18 (54.5%)	98 (43.9%)	
Periventricular leukomalacia (PVL)	22 (08.6%)	06 (18.2%)	16 (07.2%)	0.035 **
Major intraventricular hemorrhage "IVH Grade 3&4"	37 (14.5%)	14 (42.4%)	23 (10.3%)	<0.001 **
ROP that needs intervention	26 (10.2%)	03 (09.1%)	23 (10.3%)	1.000
Surgical necrotizing enterocolitis	15 (05.9%)	04 (12.1%)	11 (04.9%)	0.111
Bronchopulmonary dysplasia (BPD)	73 (28.5%)	06 (18.2%)	67 (30.0%)	0.159
Culture-positive sepsis	61 (23.8%)	09 (27.3%)	52 (23.3%)	0.619
Number of fetuses				
Singleton	27 (10.5%)	05 (15.2%)	22 (09.9%)	0.489
Triplets	148 (57.8%)	15 (45.5%)	133 (59.6%)	
Quadtruplets	56 (21.9%)	08 (24.2%)	48 (21.5%)	
Quintuplets	12 (04.7%)	03 (09.1%)	09 (04.0%)	
Sextuplets	06 (02.3%)	01 (03.0%)	05 (02.2%)	
	Mean ± SD	Mean ± SD	Mean ± SD	P-value ^‡^
Duration of ventilation	9.76 ± 19.6	18.9 ± 29.8	8.39 ± 17.3	0.004 **
Length of stays (days)	46.7 ± 38.1	22.0 ± 31.5	50.4 ± 37.7	<0.001 **
Maternal age	30.3 ± 5.66	30.6 ± 6.31	30.2 ± 5.57	0.749
Parity	1.75 ± 1.65	1.91 ± 1.94	1.73 ± 1.61	0.565
APGAR score (1 min)	5.94 ± 1.99	4.88 ± 1.39	6.09 ± 2.02	0.001 **
APGAR score (5 mins)	7.76 ± 1.68	6.67 ± 1.34	7.92 ± 1.67	<0.001 **

When measuring the relationship between the overall outcome and the patient's demographic and clinical characteristics of the patients, it was observed that the mortality rates were significantly higher among patients with gestational age between 22 and 26 weeks (p < 0.001). Moreover, we observed that increasing gestational age was associated with decreasing risk for mortality (p < 0.001). Increasing birth weight was associated with increasing risk for mortality (p = 0.002). Mortality rates were also significantly higher among patients with NSVD (p = 0.039), periventricular leukomalacia (p = 0.035), and major intraventricular hemorrhage (p < 0.001). In addition, increasing the duration of ventilation (p = 0.004) and length of stays (p < 0.001) were associated with increasing risk for mortality, but increasing one-minute APGAR score (p = 0.001) and five-minute APGAR score (p < 0.001) were associated with decreasing risk for mortality.

Taken from the significant results, univariate and multivariate regression analyses were subsequently performed (as shown in Table 2) to determine non-survivors' independent significant risk factors. Based on the results, it was observed that increasing gestational age was associated with decreasing risk for mortality, particularly between 27 and 29 weeks, with a decreased risk of 90% in both univariate (OR = 0.103; 95% CI = 0.042-0.253; p < 0.001) and multivariate regression (adjusted OR (AOR) = 0.107; 95% CI = 0.043-0.265; p < 0.001). Compared to NSVD patients, patients who underwent cesarean section had a decrease in mortality by at least 57% in the univariate analysis (OR = 0.437; 95% CI = 0.196-0.973; p = 0.043), but it did not reach statistical significance after adjustments to confounders (p = 0.058). However, patients who had PROM were at increased risk for mortality by at least 2.34 times higher in the univariate analysis (OR = 2.335; 95% CI = 1.023-5.331; p = 0.044), but it did not reach statistical significance in the multivariate analysis (p = 0.058). Patients with PVL had an increased risk for mortality by at least 2.87 times higher in the univariate analysis (OR = 2.875; 95% CI = 1.036-7.976; p = 0.043) and 3.12 times after adjustment to a regression model (AOR = 3.117; 95% CI = 1.075-9.036; p = 0.036). Patients with major IVH were 6.41 times more likely to have an increased risk for mortality in the crude analysis (OR = 6.407; 95% CI = 2.839-14.463; p < 0.001) with consistent results after adjustment to confounders (AOR = 6.630; 95% CI = 2.854-15.404; p < 0.001). Interestingly, marginal risks were observed with increasing birth weight (OR = 0.996; 95% CI = 0.993-0.999; p = 0.003 vs. AOR = 0.996; 95% CI = 0.993-0.999; p = 0.004) and increasing duration of ventilation (OR = 0.981; 95% CI = 0.966-0.995; p = 0.008 vs. AOR = 0.982; 95% CI = 0.982-0.997; p = 0.016). Increasing risks for mortality were also associated with increasing length of stays (OR = 1.048; 95% CI = 1.022-1.075; p < 0.001 vs. AOR = 1.048; 95% CI = 1.022-1.074; p < 0.001) but had decreased risk on one-minute APGAR score (OR = 0.758; 95% CI = 0.638-0.900; p = 0.002 vs. AOR = 0.747; 95% CI = 0.624-0.894; p = 0.001) and five-minute APGAR score (OR = 0.737; 95% CI = 0.622-0.874; p < 0.001 vs. AOR = 0.694; 95% CI = 0.568-0.847; p < 0.001).

**Table 2:** Univariate and multivariate regression analyses for the risk factors associated with non-survival among ELBW patients (n = 256) Adjusted with gender, maternal HTN, and DM. ** Significant at p < 0.05 level OR: odds ratio, AOR: adjusted odds ratio, NSVD: normal spontaneous vaginal delivery, PROM: premature rupture of membranes, PVL: periventricular leukomalacia, IVH: intraventricular hemorrhage

Factor	OR (95% CI)	P-value	AOR (95% CI)	P-value
Gestational age				
22-26 weeks	Ref.		Ref.	
27-29 weeks	0.103 (0.042-0.253)	<0.001 **	0.107 (0.043-0.265)	<0.001 **
30-33 weeks	0.414 (0.128-1.337)	0.140	0.411 (0.126-1.340)	0.140
Mode of delivery				
NSVD	Ref.		Ref.	
Cesarean section	0.437 (0.196-0.973)	0.043 **	0.459 (0.204-1.031)	0.059
PROM (>18 hours)	2.335 (1.023-5.331)	0.044 **	2.268 (0.972-5.294)	0.058
PVL	2.875 (1.036-7.976)	0.043 **	3.117 (1.075-9.036)	0.036 **
Major IVH (Grades 3 and 4)	6.407 (2.839-14.463)	<0.001 **	6.630 (2.854-15.404)	<0.001 **
Birth weight (grams)	0.996 (0.993-0.999)	0.003 **	0.996 (0.993-0.999)	0.004 **
Duration of ventilation	0.981 (0.966-0.995)	0.008 **	0.982 (0.968-0.997)	0.016 **
Length of stays (days)	1.048 (1.022-1.075)	<0.001 **	1.048 (1.022-1.074)	<0.001 **
APGAR score (1 min)	0.758 (0.638-0.900)	0.002 **	0.747 (0.624-0.894)	0.001 **
APGAR score (5 mins)	0.737 (0.622-0.874)	<0.001 **	0.694 (0.568-0.847)	<0.001 **

**Figure 1:**
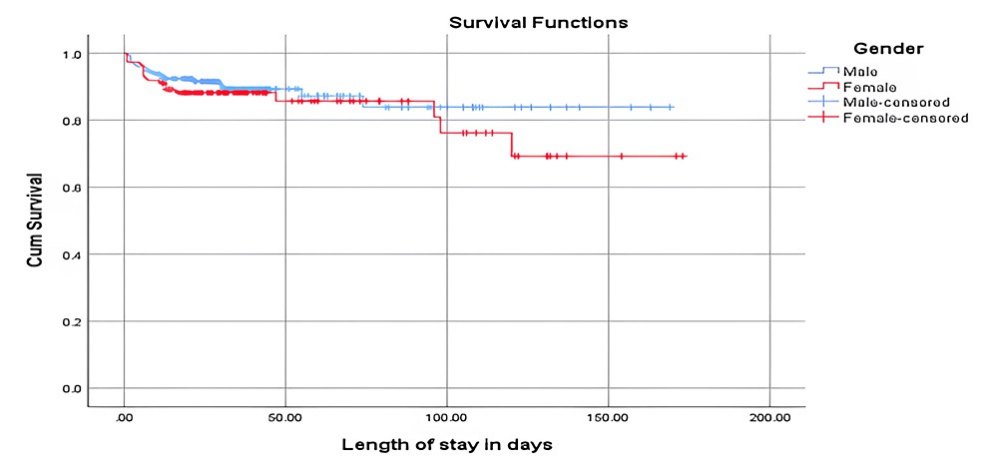
Survival plot of gender in relation to the length of stay in days. Survival functions: Y-axis: cum survival (cumulative range of survival); X-axis: length of stay in days Gender: male (blue line), female (red line), male-censored (crossed blue line), female-censored (crossed red line)

In Figure 1, the mean survival time of the male infants was 146.6 (standard error 5.65), while the mean survival time of the female infants was 139.3 (standard error 7.86). The overall mean survival time was 144.7 (standard error 5.00). The analysis revealed that the difference was not statistically significant based on the log rank (Mantel-Cox) (p = 0.375).

**Figure 2:**
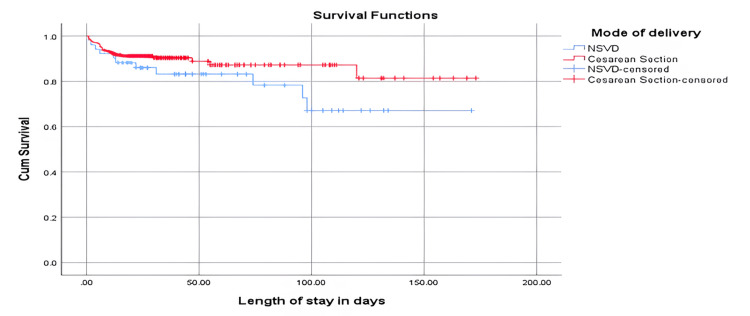
Survival plot of the method of delivery in relation to the length of stay in days Survival functions: Y-axis: cum survival (cumulative range of survival); X-axis: length of stay in days Mode of delivery: NSVD: normal spontaneous vaginal delivery (blue line), cesarean section (red line), NSVD-censored: normal spontaneous vaginal delivery-censored (crossed blue line), cesarean section-censored (crossed red line)

In Figure 2, the NSVD mean survival time was 131.3 (standard error 10.357), while the cesarean mean survival time was 150.2 (standard error 5.157). The analysis revealed that the difference was not statistically significant based on the log rank (Mantel-Cox) (p = 0.114).

**Figure 3:**
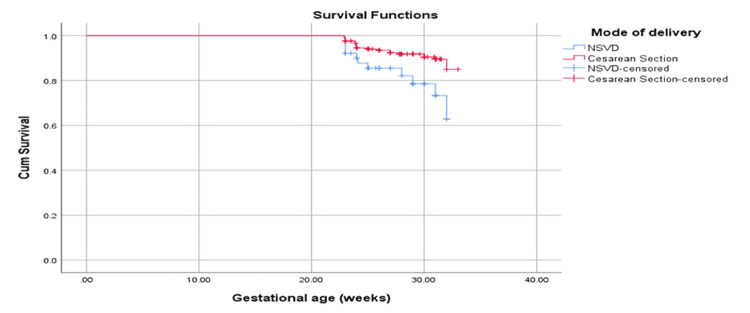
Survival plot of the method of delivery in relation to gestational weeks Survival functions: Y-axis: cum survival (cumulative range of survival); X-axis: gestational age (weeks) Mode of delivery: NSVD: normal spontaneous vaginal delivery (blue line), cesarean section (red line), NSVD-censored: normal spontaneous vaginal delivery-censored (crossed blue line), cesarean section-censored (crossed red line)

When measuring the survival time according to gestational weeks in relation to the method of delivery (Figure 3), it was found that the NSVD mean survival time was 30.5 weeks (standard error 0.464), while cesarean mean survival time was 32.2 weeks (standard error 0.171). The overall mean survival time was 32.01 (standard error 0.170). The analysis revealed that the difference was statistically significant based on the log rank (Mantel-Cox) (p = 0.008).

## Discussion

Neonatal death is a major public health concern worldwide. Although there was a decreasing trend of infant mortality appearing throughout the globe, changes in the incidence of neonatal death have befallen much slower [9]. In this study, the prevalence of neonatal death was 12.9%. This is consistent with the study done in Iran, which showed a prevalence of 14.4% [10]. A similar result has been documented in India with neonatal mortality rates of 20.5% [11]. However, in a study done in Riyadh [2] and in Thailand [12], incidences of neonatal death were much higher at 41% and 36.9%, respectively. On the other hand, our study noted a survival-discharge rate of 87.1%, which is consistent with previous studies [13].

This study's most significant risk factors for neonatal death were PROM, PVL, major IVH, and longer hospital stays. In Japan, Ogawa et al. (2013) documented that normal delivery, nonvertex presentation, and placental abruption were significantly associated with neonatal death [14], while Alhamad et al. (2022) indicated that mortality was primarily associated with lower gestational ages and no platelet transfusions [7]. Notwithstanding these reports, Gupta et al. (2021) documented that ELBW, respiratory distress syndrome requiring surfactant therapy, hyperglycemia, and severe intraventricular hemorrhage were neonatal death significant risk factors [11].

Likewise, we detected a marginal association between survival in terms of birth weight and duration of ventilation. This is comparable to the study conducted by Jia et al. (2022), which reported that weight, specialized hospitals, and regional economic developments were closely associated with increased survival [15]. However, in a study conducted by AlQurashi et al. (2021), female infants with a gestational age of ≤26 weeks and birth weight ≤750 grams had a higher chance of survival compared to males, but the pairwise comparison yielded insignificant results (p > 0.05) [1].

Moreover, better survival depended mainly on increasing APGAR score and cesarean section. This mirrored the results of Moghaddam and Aghaali (2015), who found that an APGAR score of more than 5 at the first minute and more than 7 at the fifth minute were associated with better survival after hospital discharge. Moreover, they reported that increased birth weight and gestational age were correlated with decreased mortality. In South Africa, birth weight was the main predictor of survival [13]. Supplemental predictors were gender, birth before arrival at the hospital (BBA), hypotension, necrotizing enterocolitis (NEC), and continuous positive nasal airway pressure (NCPAP). A similar report in Japan found a significant association between the survival rate in cesarean and vaginal delivery during 24-31 weeks of gestation [14]. In addition, infants with a birth weight >400 g on cesarean delivery significantly contributed to the survival rate of ELBW infants compared to vaginal delivery.

The pattern of neonatal comorbidities was comparable with other studies. For instance, Altirkawi et al. (2021) documented that the prominent morbidity was respiratory distress syndrome (88.5%) [8]. Other forms of morbidities were less common, including retinopathy of prematurity (28.3%), patent ductus arteriosus (PDA) (27.9%), and sepsis/meningitis (25.6%). This was echoed by the reports of Kiatchoosakun et al. [12]. Respiratory distress syndrome (70.7%), neonatal jaundice (66.7%), and sepsis (60.4%) were the three topmost frequent comorbidities diagnosed among ELBW infants. In our study, however, bronchopulmonary dysplasia (28.5%), culture-positive sepsis (23.8%), major IVH (14.5%), ROP (10.2%), and periventricular leukomalacia (8.6%) were the most common complications detected in ELBW infants.

Using the Kaplan-Meier method, we assessed the survival of ELBW infants concerning length of hospital stay and gestational age. According to our results, the differences in survival between the gender and the delivery method in terms of the length of hospital stay yielded similar results (p > 0.05). However, in terms of gestational age (weeks), we noted a significant difference in survival time among infants who were delivered by cesarean method (p = 0.008). This contradicted the reports of Moghaddam and Aghaali [10], wherein the mortality rate by normal vaginal delivery born in LBW infants was greater than in cesarean section delivery; however, cesarean section delivery had higher risk mortality in VLBW and ELBW infants than that of normal vaginal delivery.

Our study has some strengths and limitations. First, this was a retrospective study; charts and medical records were used to gather data for both moms and infants. Second, the experience of a single institution is reflected in this data. As such, caution must be used when extrapolating and interpreting our data. Lastly, all instances with ELBW born within a four-year period were included in our case-control research. It is crucial to remember that our study lacked a sample size or power estimate, which we recognize as a drawback in our methodology.

The purpose of this study was to assess the survival rates of infants with ELBWs and any potential prenatal, perinatal, and postnatal risk factors that may be associated with mortality. These findings, in our opinion, are helpful in advising parents when a suspected delivery of an ELBW occurs.

## Conclusions

Thirteen percent of the ELBW neonates died before discharge. Non-survival varied significantly by PROM, PVL, major IVH, and longer hospital stays. However, decreasing risk for non-survival differed significantly by cesarean section, increasing gestational age, and APGAR scores. It is interesting to know that birth weight and the duration of ventilation were marginally dependent on non-survival. A more desirable health outcome necessitates strategies to improve understanding of the issues and proper execution of the solution through an evidence-based approach with emphasis on improving NICU facilities, including early referral of pregnant women and neonates who are at high risk, which could lead to the future decline of morbidities and mortalities among neonates.
